# Ponderomotive plasma lenses for holography by Gaussian beams

**DOI:** 10.1038/s41598-026-41214-x

**Published:** 2026-02-27

**Authors:** Sima Alilou, Laya Shahrassai, Samad Sobhanian

**Affiliations:** Faculty of Physics, University of Tabriz, Tabriz, 009841 Iran

**Keywords:** Ponderomotive force, Plasma lenses, Holography, Laser-plasma interaction, Optics and photonics, Physics

## Abstract

Plasma-based optical elements, including ponderomotive plasma lenses, offer distinctive capabilities for energy steering, localization, and high-sensitivity imaging in dynamic media. In this work, we investigate the application of ponderomotive plasma lenses to holography by utilizing the interference of two Gaussian laser beams. The analysis focuses on how the collective plasma response to spatial intensity gradients leads to refractive index modulation and dynamic wavefront control. By explicitly defining the holographic geometry and distinguishing between the reference and sample beams, we show that the achievable image resolution is strongly influenced by the transverse properties of both Gaussian inputs. In particular, the beam widths play a central role in determining the coherence, stability, and spatial-frequency content of the interference pattern. Appropriate adjustment of the individual beam waists improves fringe quality and phase sensitivity, resulting in enhanced spatial resolution and more accurate reconstruction of the three-dimensional refractive-index distribution. The present study is theoretical and employs laser parameters that are accessible in laboratory-scale systems, providing a physically realistic basis for plasma-based holography using standard solid-state laser sources.

## Introduction

Plasma-based optical elements leverage the nonlinear interaction between electromagnetic fields and a partially ionized medium^1^. The ponderomotive force, arising from spatial gradients of field intensity, induces collective motion and refractive-index modulation, enabling controllable focusing and steering of energy without mechanical contact^2,3^. Plasma holography extends conventional holographic concepts to plasmas, using interference between a reference field and a field modified by the plasma to encode spatial information about the plasma’s optical properties and charge-density distribution^4–6^. The dual themes—ponderomotive plasma lenses and plasma holography—offer potential advantages in energy concentration, material efficiency, thermal management, and high-sensitivity metrology, while presenting challenges related to stability, noise, and environmental fluctuations^7–9^.

The ponderomotive force is the gradient of the intensity, $F_{p} \propto - \nabla I$, where I is the local intensity of the electromagnetic field^10^. This force redistributes plasma electrons, creating density gradients that modify the local refractive index^11^. Nonlinear focusing arises because the redistribution changes the phase velocity across the beam profile, yielding convergence or steering of the electromagnetic energy^12^. The mechanism is mediated by collective plasma dynamics rather than solid boundaries, offering potential advantages for high-flux or fast-replenishment regimes^13^.

In the present holographic configuration, the role of the ponderomotive force is not limited to particle displacement but extends to a direct modulation of the plasma refractive index. Spatial variations of the optical intensity generated by the interference of the reference and sample Gaussian beams exert a ponderomotive force on the electron population, driving electrons away from high-intensity regions. This redistribution leads to local changes in the electron density, and consequently alters the refractive index through its intrinsic density dependence. As a result, the plasma does not merely act as a passive scattering medium; instead, its refractive index profile dynamically follows the intensity landscape imposed by the interfering fields. The recorded hologram therefore reflects a refractive-index modulation induced by the ponderomotive mechanism, which forms the physical basis for phase and amplitude encoding in the reconstructed plasma structure.

As intensity of gaussian laser beams increases, nonlinearity can introduce adverse effects (e.g., nonlinear phase distortions, turbulence, plasma heating) that reduce net focusing efficiency^14^. Nonetheless, a regime exists where useful performance remains acceptable, enabling operations in high-energy applications under appropriate constraints^15^. Plasma holography records the interference between a reference optical field and a field that has interacted with or been scattered by the plasma sample^16^. The resulting interference pattern encodes spatial variations in the plasma’s refractive index and charge-density distribution. The hologram maps the spatial-frequency content of the plasma’s optical response, enabling three-dimensional reconstruction of its refractive-index profile. Time-resolved interference data can capture dynamical plasma responses, offering insights into transient electrostatic, electromagnetic, or density fluctuations. Variations in local electron density produce phase and amplitude changes in the interacting light, which appear as fringes in the recorded hologram^17^. The plasma’s dispersive and nonlinear properties can enhance sensitivity to thin or multi-layered structures in certain regimes^18^.

In plasma lenses, energy concentration benefits at moderate intensities may give way to efficiency decline at higher input energies due to nonlinear distortions, wave-front aberrations, and elevated plasma temperatures^19^. Identify an operational window where energy concentration remains high with acceptable efficiency, enabling high-energy applications such as pulsed power delivery, laser-plasma acceleration, or compact beam-steering devices.

Compared to conventional solid-state optical lenses, plasma-based lenses offer several distinctive features that are particularly relevant in holographic configurations. Because the refractive index in plasma is governed by collective charge dynamics rather than fixed material boundaries, plasma lenses are not subject to permanent damage or thermal degradation under high optical intensities. In addition, the refractive-index profile can be continuously modified through changes in the driving laser parameters or plasma conditions, allowing dynamic control of wave-front curvature without mechanical adjustment. In the context of holography, this adaptability enables the lensing action and the holographic phase encoding to arise from the same optical interaction, rather than from separate passive elements. As a result, plasma-based lenses provide a flexible platform for energy steering and phase-sensitive imaging in regimes where conventional optics become inefficient or impractical^20^.

A notable advantage is reduced reliance on solid materials, potentially lowering material consumption and enabling rapid, repeatable operation. Thermal load on surrounding structures can be mitigated by the plasma’s ability to localize energy deposition and by strategic engineering of plasma parameters and geometry. Plasma fluctuations, environmental vibrations, and detector noise pose limitations for both plasma lensing and holography.

Although the analysis in this work is formulated for Gaussian beams, the underlying ponderomotive mechanism is not inherently limited to this class of optical fields. The ponderomotive force is governed by spatial gradients of the optical intensity, and therefore any beam profile that produces a structured transverse intensity distribution can, in principle, induce a corresponding modulation of the plasma refractive index. For non-Gaussian modes such as Bessel or Laguerre–Gaussian beams, the resulting density redistribution would follow the characteristic ringed or vortex-like intensity patterns of these fields. While the detailed form of the Laplacian of the intensity would differ from the Gaussian case, the holographic encoding would still arise from the same physical coupling between intensity gradients, electron density, and refractive index. In the present study, Gaussian beams are adopted to maintain analytical transparency and to isolate the essential features of the holographic response^21^. Unlike previous studies that investigated ponderomotive plasma lenses primarily for focusing or beam steering, the present work establishes a direct and quantitative framework connecting ponderomotive refractive-index modulation to holographic phase encoding and image resolution using two independently controlled Gaussian beams.

In this work, we investigate the feasibility and performance of ponderomotive plasma lenses for holography, with particular emphasis on the role of the optical frequency (or equivalently the wave number) of the interacting Gaussian beams. Here, the term “frequency” refers to the spectral and spatial-frequency characteristics of the laser fields that govern interference fringe formation and phase evolution. By analyzing how variations in beam frequency and wave number affect the resulting intensity gradients, we examine their influence on ponderomotive refractive-index modulation, energy steering, and holographic fidelity in dynamic plasma media.

## Calculating

Two common approaches to holography using two laser beams are: (1) two independent lasers, in which Laser A serves as the reference beam and Laser B serves as the object beam, with careful synchronization of phase and frequency and (2) a single laser divided into reference and sample paths using a beam splitter and mirrors, providing control over the relative path length and angle^22^.

In this study, the holographic configuration based on two independent laser beams is adopted rather than a beam-splitter arrangement. This choice is motivated by the need for independent control over the parameters of the reference and sample fields in a plasma environment. In the presence of ponderomotive interactions, the plasma response can introduce local phase shifts and amplitude modulations that are sensitive to beam width, intensity, and frequency. Using two independently adjustable Gaussian beams allows these parameters to be tuned separately, facilitating a clearer assessment of their influence on the holographic interference pattern. Although beam-splitter-based schemes provide inherent phase coherence, they also impose stricter constraints on optical path stability and symmetry, which may limit flexibility when plasma-induced distortions are present. For the purposes of the present analysis, the two-laser approach offers a more adaptable framework for examining ponderomotive plasma lensing effects in holography.

Here we have used the first method. The total field as the sum of Gaussian reference and Gaussian sample is^23^:

1 $$E_{tot} (r,z) = E_{r} (r,z) + E_{s} (r,z)$$

The measured intensity (optical signal) as the squared magnitude of the total field is:

2 $$\left| {E_{tot} (r,z)} \right|^{2} = \left| {E_{r} (r,z) + E_{s} (r,z)} \right|^{2} = I(r,z)$$

3 $$I(r,z) = \left| {E_{r} } \right|^{2} + \left| {E_{s} } \right|^{2} + 2{\mathrm{Re}} \{ E_{r} E_{s}^{*} \}$$

The Gaussian field used for reference is defined as follows^24^:

4 $$E_{r} (r,z) = E_{0r} \frac{{\omega_{0r} (z)}}{{\omega_{r} (z)}}e^{{\left( { - \frac{{r^{2} }}{{\omega_{r}^{2} (z)}} + i\phi_{r} (r,z)} \right)}} ,$$

and the sample field is:

5 $$E_{s} (r,z) = E_{0s} \frac{{\omega_{0s} (z)}}{{\omega_{s} (z)}}e^{{\left( { - \frac{{r^{2} }}{{\omega_{s}^{2} (z)}} + i\phi_{s} (r,z)} \right)}} ,$$

where $E_{0r}$ and $E_{0s}$ are the on-axis amplitudes, $\omega_{r} (z)$ and $\omega_{s} (z)$ are the beam radii at position z, $\omega_{0r} (z)$ and $\omega_{0s} (z)$ are the waist-related factors, and $\phi_{r} (r,z)$ are the Gouy-phase–modified phases associated with the reference and sample beams, respectively^25^.

The interference intensity $I(r,z)$ at position (r, z) is the sum of the reference sample and the interference intensities, given explicitly by:

6 $$I(r,z) = I_{r} (r,z) + I_{s} (r,z) + I_{{\mathrm{int}}} (r,z)$$

7 $$\begin{aligned} I(r,z) & = E_{{_{0r} }}^{2} \frac{{\omega_{{_{0r} }}^{2} (z)}}{{\omega_{{_{r} }}^{2} (z)}}e^{{\left( { - \frac{{2r^{2} }}{{\omega_{r}^{2} (z)}}} \right)}} + E_{{_{0s} }}^{2} \frac{{\omega_{0s}^{2} (z)}}{{\omega_{s}^{2} (z)}}e^{{\left( { - \frac{{2r^{2} }}{{\omega_{s}^{2} (z)}}} \right)}} \\ & \quad + 2E_{0r} E_{0s} \frac{{\omega_{0r} (z)\omega_{0s} (z)}}{{\omega_{r} (z)\omega_{s} (z)}}e^{{ - \left( {\frac{{r^{2} }}{{\omega_{r}^{2} (z)}} + \frac{{r^{2} }}{{\omega_{s}^{2} (z)}}} \right)}} \cos (\Delta \phi (r,z)) \\ \end{aligned}$$

Since the optical intensity is a real-valued quantity, it is obtained from the product of the total field and its complex conjugate. Accordingly, the interference contribution is expressed through the cosine of the relative phase difference between the reference and sample fields, ensuring that no explicit complex terms appear in the final intensity distribution.

where $\Delta \phi (r,z) = \phi_{r} (r,z) - \phi_{s} (r,z)$ is the phase difference between the reference and sample fields. The holographic response considered here is sensitive to the relative phase stability between the reference and sample Gaussian beams, primarily through its influence on the visibility of the interference fringes. Phase fluctuations introduce temporal variations in the interference term, which tend to reduce fringe contrast when averaged over the observation time. However, such instabilities do not directly suppress the ponderomotive interaction itself, since the latter is governed by the spatial gradients of the optical intensity. In the present analysis, phase variations are assumed to be either slow compared to the measurement timescale or sufficiently stable to preserve the dominant interference structure. Under these conditions, the essential features of the holographic modulation remain accessible, while moderate phase noise manifests mainly as a reduction in contrast rather than a qualitative change in the reconstructed plasma response. In the rest of the article, we have omitted the variation of the phase difference for simplicity of calculations. This expression encapsulates the Gaussian envelopes of both beams and the interference fringe term that modulates the intensity via the relative phase, and it underpins the holographic reconstruction of plasma-related phase and amplitude distributions. According to the initial assumption, the charge density in the sample is directly proportional to the Laplacian of the intensity distribution^26^:

8 $$\rho (r,z) \propto - \nabla^{2} I(r,z)$$

where $I(r,z)$ is the field intensity. The first step is to compute the Laplacian of I in cylindrical coordinates, assuming radial symmetry (only r is relevant) and a uniform z-direction (flat in z). For a radial function I(r), the Laplacian reduces to $\nabla^{2} I(r) = \frac{1}{r}\frac{\partial }{\partial r}(r\frac{\partial I}{{\partial r}})$. This expression explicitly shows how the radial curvature of the intensity profile governs the ponderomotive driving term that shapes the plasma density distribution. If desired, the full 3D form can be reinstated by incorporating z-dependence, but under the present assumptions the above 1D radial form suffices. To explain how the Laplacian of the intensity distribution can be utilized for analyzing charge density and ponderomotive force, we must review some fundamental relationships. In optical structures, the electro optical field and the electric field within the sample significantly influence the distribution of electric charge. The oscillatory and interference effects of optical fields create controllable structures in optical samples, including plasma holograms. Ponderomotive Force is the force exerted by optical fields on electric charges over time and space. This force is based on the variations in intensity and the charge distribution within the sample. This relationship indicates that regions where $\rho (r,z) \propto - \nabla^{2} I(r,z)$ is large and positive harbor more negative charges, and vice versa. Thus, by calculating the Laplacian of the intensity distribution, we can analyze and predict the charge distribution, and consequently, the forces acting on the sample and its internal structures based on these relationships. This approach serves as a powerful tool in the design of optical holograms and the control of optical structures, including plasma holograms. In plasma, the connection between the field and charge distribution is derived from Poisson’s equation^27^:

9 $$\nabla^{2} \phi (r,z) = \frac{\rho (r,z)}{{\varepsilon_{0} }}$$

where $\phi (r,z)$, $\rho (r,z)$ and $\varepsilon_{0}$ are electric potential, charge density and permittivity of free space, respectively. The optical field, in this context, arises due to the distribution of existing charges; thus $E = - \nabla \phi$. The intensity of the optical field $I(r,z)$ is directly related to the electric field, following a general relationship $I(r,z) \propto E^{2}$, In simplified assumptions, variations in field intensity align with changes in potential and charge from the initial assumption $F_{p} \propto \nabla I(r,z)$.Since the force acts on the particles, and the particles are in equilibrium, the ponderomotive force must balance with other forces and the charge distribution^25^. In a stable scenario, the charge density must relate to the field distribution as follows:

10 $$\rho (r,z) \propto - \nabla .F_{p} \to \rho (r,z) \propto - \nabla^{2} I(r,z)$$

This relationship demonstrates that the charge density is directly dependent on the second derivative of the intensity, resulting from the ponderomotive force effect that displaces particles based on the gradient of the field at various radii and heights.

The use of a Laplacian-based description of charge density in plasma is accompanied by both analytical and computational challenges when dynamic effects are considered. In realistic plasma environments, the charge distribution evolves in time and is coupled to variations in temperature, field amplitude, and particle motion, leading to nonlinear feedback between the optical field and the plasma response. Accounting for such dynamics generally requires solving coupled fluid or kinetic equations together with Maxwell’s equations, which rapidly increases computational complexity. From an analytical perspective, closed-form expressions become difficult to obtain once spatial symmetry is relaxed or temporal fluctuations are included. In the present study, these difficulties are mitigated by restricting the analysis to a radially symmetric and quasi-static regime, where the Laplacian of the intensity provides a reasonable representation of the dominant ponderomotive contribution to the charge density. This approximation allows the essential holographic features to be examined without obscuring the physical interpretation.

In two-dimensional space, the Fourier transform *F{f(r)}* of a radially symmetric function *f(r)* where $r = \sqrt {x^{2} + y^{2} }$ is given by:

11 $$F\{ f(r)\} (k_{r} ) = 2\pi \int\limits_{0}^{\infty } {f(r)J_{0} (k_{r} r)} rdr$$

where $J_{0}$ is the Bessel function of the first kind, order zero, $k_{r}$ is the radial wave number. Suppose $f(r) = e^{{ - \frac{{r^{2} }}{{\omega^{2} }}}}$, This is a radially symmetric Gaussian function. Its Fourier transform in 2D (also called the Hankel transform of order zero) can be derived using integral identities involving Bessel functions. The key integral identity used is:

12 $$\int\limits_{0}^{\infty } {e^{{ - \alpha r^{2} }} J_{0} (\beta r)} rdr = \frac{1}{2\alpha }e^{{ - \frac{{\beta^{2} }}{4\alpha }}} .$$

13 $$F\{ f(r)\} = 2\pi \frac{1}{2\alpha }e^{{ - \frac{{\beta^{2} }}{4\alpha }}} \to F\{ f(r)\} = \pi \omega^{2} e^{{ - \frac{{\omega^{2} }}{4}k_{r}^{2} }}$$

This derivation relies on standard integral transforms and identities from the theory of Fourier transforms of radially symmetric functions. The charge density $\rho (r,z)$ includes a Gaussian component combined with a polynomial term, expressed as:

14 $$\begin{aligned} \rho (r,z) & \propto \left[ {\frac{8}{{\omega_{r}^{2} (z)}}I_{r} (r,z) - \frac{{16r^{2} }}{{\omega_{r}^{4} (z)}}I_{r} (r,z)} \right] \\ & \quad + \left[ {\frac{8}{{\omega_{s}^{2} (z)}}I_{s} (r,z) - \frac{{16r^{2} }}{{\omega_{s}^{4} (z)}}I_{s} (r,z)} \right] \\ & \quad + \left[ {I_{{\mathrm{int}}} (r,z) - \left( {\frac{{16r^{2} }}{{\omega_{r}^{4} (z)}} + \frac{{16r^{2} }}{{\omega_{s}^{4} (z)}}} \right)I_{{\mathrm{int}}} (r,z)\left( {\frac{8}{{\omega_{s}^{2} (z)}} + \frac{8}{{\omega_{r}^{2} (z)}}} \right)} \right] \\ \end{aligned}$$

The function $H(k)$ defined as the Laplacian of the intensity I(r) demonstrates the holographic properties:

15 $$\begin{aligned} H(k) & = \left| {E_{0r} } \right|^{2} (6\pi - \pi k_{r}^{2} )e^{{ - \frac{{\omega_{r}^{2} k_{r}^{2} }}{4}}} + \left| {E_{0s} } \right|^{2} (6\pi - \pi k_{s}^{2} )e^{{ - \frac{{\omega_{s}^{2} k_{s}^{2} }}{4}}} \\ & \quad + 2\left| {E_{0r} } \right|\left| {E_{0s} } \right|(12\pi - \pi (k_{r}^{2} + k_{s}^{2} )e^{{ - \frac{{(\omega_{r}^{2} + \omega_{s}^{2} )k_{{\mathrm{int}}}^{2} }}{4}}} \\ \end{aligned}$$

Refractive-index modulations due to ponderomotive effects can be experimentally observed by utilizing optical diagnostics such as probe beam imaging or interferometry. In a laboratory setup, a low-power probe beam can be passed through the plasma and its transmission or deflection can be measured. The deflection of the probe beam is directly related to the spatial variation in the refractive index caused by the ponderomotive force. The refractive-index modulation, $\Delta n(r)$, can be quantified using Snell’s Law, which relates the angle of refraction to the refractive index:

$$\Delta \theta = \frac{\Delta n(r)}{{n_{0} }}$$

where $n_{0}$ is the background refractive index of the plasma, and $\Delta \theta$ is the angular shift of the probe beam caused by the refractive index gradient. By measuring the deflection angle, we can estimate the local refractive-index modulation and compare it with the theoretical predictions. An interferometer, such as a Mach–Zehnder or Michelson interferometer, can split the probe beam into two arms, with one beam passing through the plasma and the other through a reference medium. The interference pattern formed by the two beams will reflect the phase shift induced by the refractive index variations. The visibility of the fringes can be directly related to the spatial and temporal modulation of the refractive index, providing a quantitative measure of the ponderomotive effect. The phase shift $\Delta \phi$ can be obtained from the intensity of the interference fringes:

$$I = I_{0} (1 + \cos (\Delta \phi ))$$

where $\Delta \phi$ is the phase difference between the reference and the sample beams. A shift in the interference fringes indicates a change in the refractive index, which can then be compared to the theoretical predictions. The reconstructed 3D refractive-index profile can be compared to the theoretical predictions by performing a phase retrieval algorithm, which uses the interference data to reconstruct the spatial distribution of the refractive index:

$$n(x,y,z) = {\mathrm{Re}} \left( {\frac{{I_{reconstructed} (x,y,z)}}{{I_{backgrand} }}} \right)$$

where $I_{reconstructed} (x,y,z)$ is the reconstructed intensity profile, and $I_{backgrand}$ is the intensity in the absence of plasma modulation.

To illustrate the feasibility of experimental validation, consider a low-density plasma with a background refractive index $n_{0} \approx 0.9995$ . A probe beam passing through this plasma can experience angular deflections of approximately $0.5^{^\circ }$, corresponding to refractive-index modulations $\Delta n(r) \sim 8.7 \times 10^{ - 3}$ . In a Mach–Zehnder interferometer, these modulations produce observable phase shifts $(\Delta \phi \sim 0.055rad)$ in the interference fringes, which can be used to reconstruct the 3D refractive-index profile via phase retrieval algorithms. Furthermore, time-resolved pump-probe measurements with ultrafast laser pulses enable observation of the dynamic evolution of the refractive index, providing a direct comparison with theoretical predictions for both static and transient plasma responses. These approaches demonstrate the practical feasibility of validating the predicted refractive-index modulations and holographic reconstructions, thereby strengthening the connection between theory and experiment^28^.

We study in this research the variation of this function H(K) with lasers wave numbers. The laser parameters adopted in this study are consistent with those commonly available in laboratory-scale solid-state laser systems. In practice, Nd:YAG lasers operating at a wavelength of 1064 nm can deliver either continuous-wave or pulsed radiation with output powers ranging from a few watts to several tens of watts. For Gaussian beam waists on the order of 20–50 μm, these operating conditions correspond to peak intensities in the range of ${10}^{13}$–${10}^{15}{\hspace{0.17em}}\mathrm{W}/{\mathrm{m}}^{2}$, which are sufficient to induce measurable ponderomotive effects in plasma while remaining below the relativistic regime. Higher peak intensities can be readily achieved using Q-switched operation, which is widely employed in Nd:YAG systems to generate short, high-energy pulses and has been demonstrated in holographic and plasma-related laser configurations^29^.

## Discussion

**Fig. 1:**
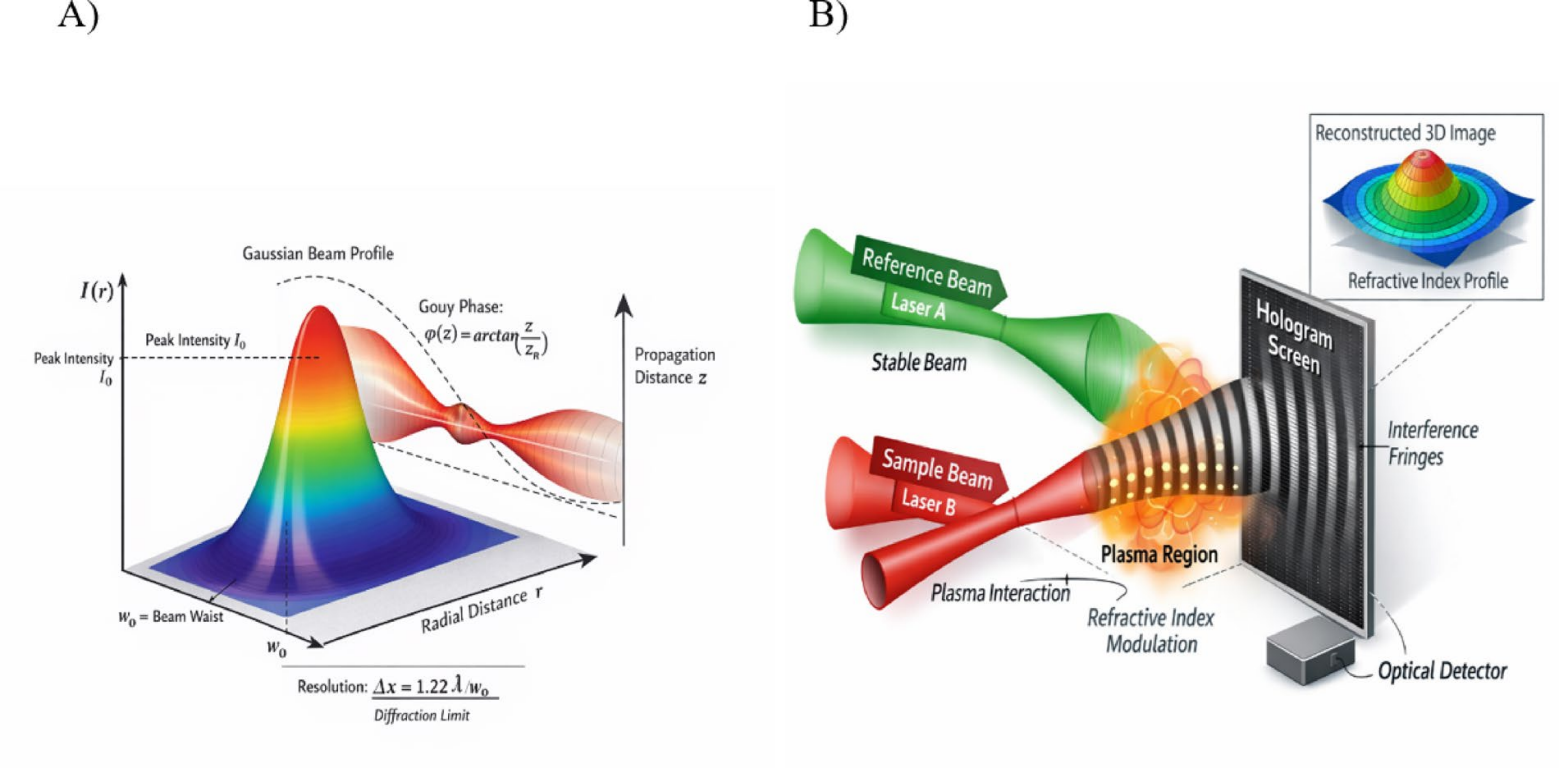
Schematic three-dimensional (**A**) representation of a Gaussian laser beam propagating in plasma (**B**) illustration of the holographic configuration based on two Gaussian laser beams interacting with a plasma medium.

The stability of the interference pattern in plasma-based holography is strongly influenced by a limited set of plasma parameters, with electron density and temperature playing the dominant roles. Spatial or temporal fluctuations in electron density directly modify the local refractive index, introducing phase variations that can distort the interference fringes. Electron temperature affects stability more indirectly, through enhanced thermal motion and increased collisional effects, which contribute to phase noise and reduce fringe visibility. Additional factors such as density gradients and weak plasma turbulence may further perturb the interference pattern, particularly over longer observation times. In the present configuration, two Gaussian laser beams are employed with a well-defined geometric arrangement in Fig. 1. One beam is designated as the reference beam, providing a stable phase baseline, while the second beam serves as the sample (object) beam and interacts with the plasma, carrying information about the induced refractive-index modulation. The two beams overlap within the plasma region under a lateral interference geometry, allowing the formation of spatial interference fringes across the transverse plane. This arrangement enables the holographic encoding of plasma-induced phase variations through the resulting intensity pattern. Although both beams contribute to the total intensity distribution, only the sample beam is assumed to carry plasma-modified information, while the reference beam remains unperturbed.

In Fig. 1, the x-axis represents the radial coordinate $r$, indicating the distance from the beam center, which is essential for characterizing the spatial intensity distribution of the laser in the plasma. The y-axis corresponds to the intensity $I(r)$, which follows a Gaussian profile, $I(r) = I_{0} e^{{( - \frac{{r^{2} }}{{w^{2} }})}}$ , where $I_{0}$ is the peak intensity and $w$ is the beam waist at the focus. The z-axis denotes the propagation distance, along which the beam acquires a Gouy phase shift, $\phi (z) = {\mathrm{Arctan}} (z/z_{R} )$, with $z_{R} = {{\pi w_{0}^{2} } \mathord{\left/ {\vphantom {{\pi w_{0}^{2} } \lambda }} \right. \kern-0pt} \lambda }$ representing the Rayleigh range. The beam waist ${w}_{0}$ defines the radius where the intensity falls to $\frac{1}{e}$ of its maximum, which is critical for determining the diffraction-limited resolution of the system, given by the Rayleigh criterion $\Delta x = 1.22\lambda /w_{0}$. Moreover, the Gouy phase influences the curvature of the beam’s wavefront, affecting the resulting interference patterns, particularly at extended propagation distances.

**Fig. 2:**
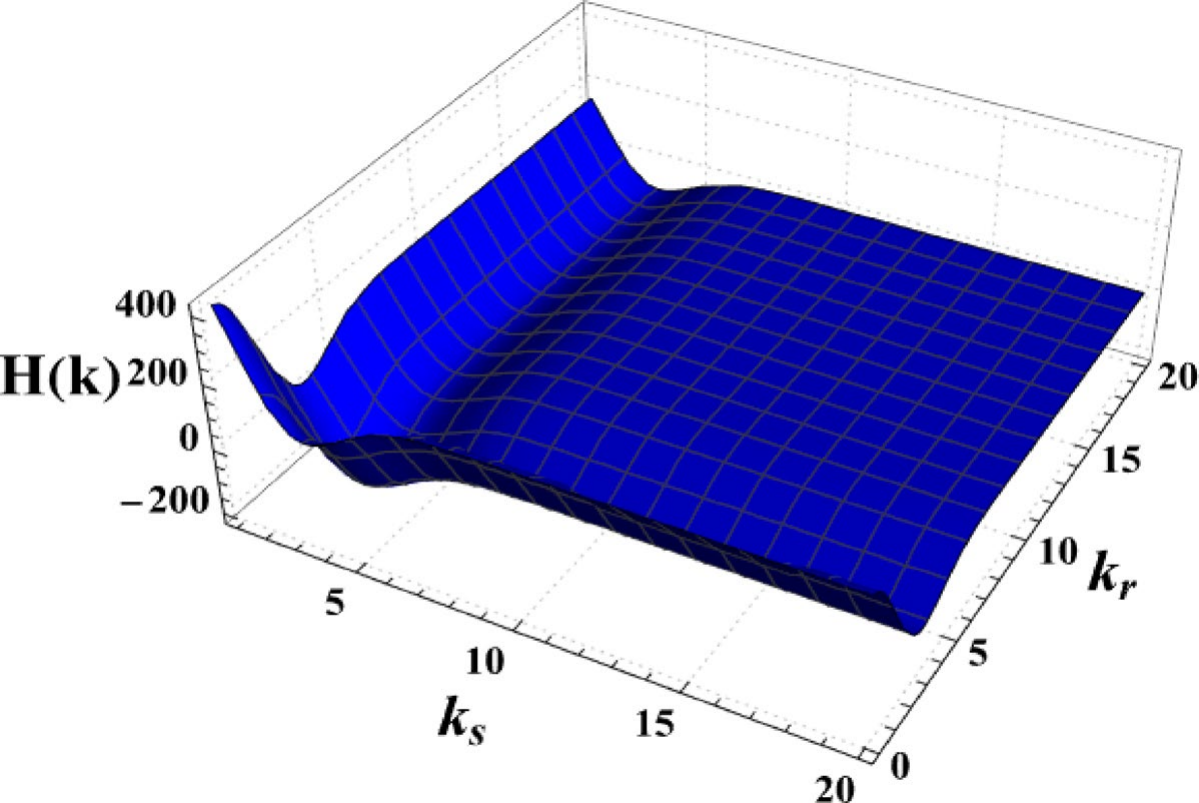
Three-dimensional holographic response as a function of the wave numbers $k_{r}$ and $k_{s}$ of the reference and sample Gaussian beams, respectively.

The spatial structure of the resulting hologram reflects the transverse interference fringes produced by the two Gaussian beams, whose spatial-frequency components are determined by the beam widths and relative wave numbers. The ponderomotively induced refractive-index modulation follows this structured intensity distribution, allowing the three-dimensional plasma response to be inferred from the recorded hologram through standard reconstruction procedures. In this sense, the three-dimensional information arises from the combined spatial-frequency content of the interference pattern rather than from a single projection. Figure 2 shows 3D holographic diagram in terms of $k_{r}$ and $k_{s}$. By observing the 3D holographic diagram in terms of $k_{r}$ and $k_{s}$, the holographic amplitude reflects the spatial-frequency content of the interference pattern formed by the two beams. At lower wave numbers, destructive interference dominates, leading to a rapid decrease in holographic amplitude, whereas increasing wave number enhances constructive interference and stabilizes the holographic response. This behavior demonstrates the sensitivity of plasma-induced refractive-index modulation to the spectral and spatial-frequency characteristics of the interacting optical fields.

**Fig. 3:**
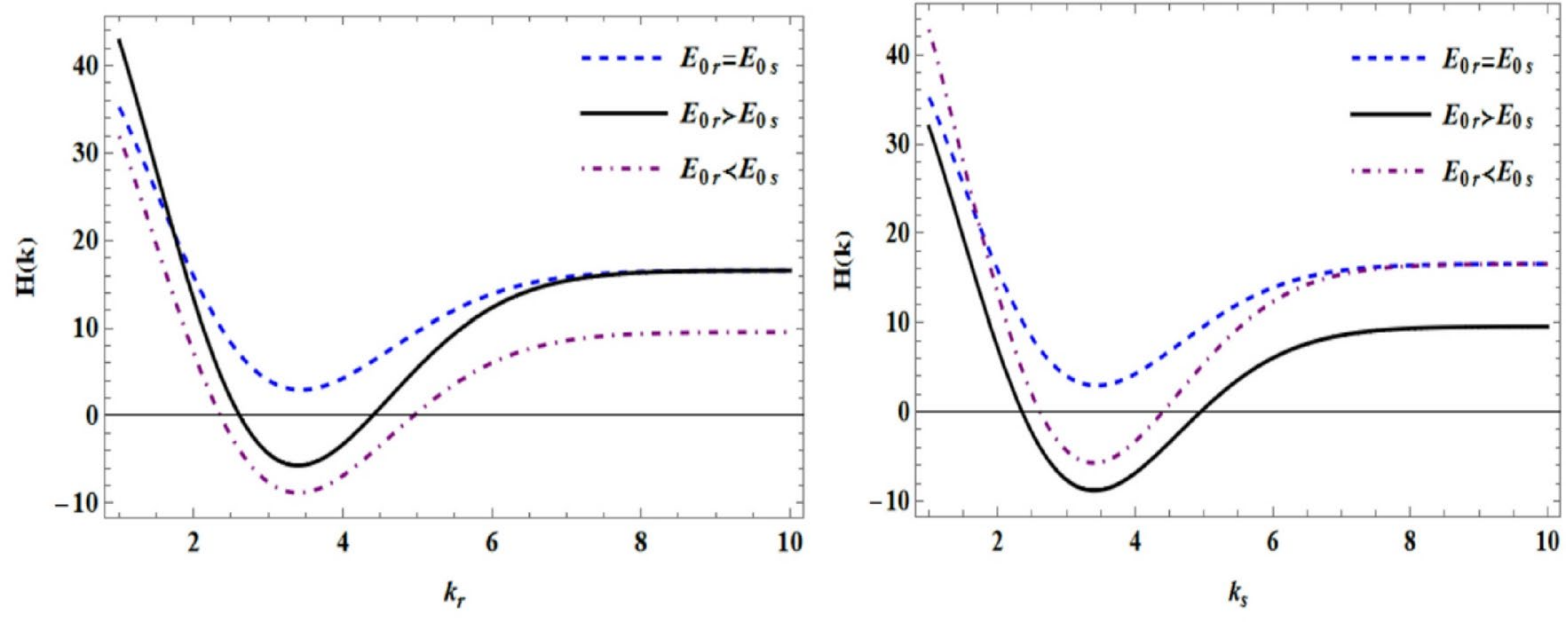
Holographic (quality of the interference fringes) diagram in terms of $k_{r}$ and $k_{s}$ with different amplitudes.

By comparing different amplitudes for the waves as function of $k_{r}$ and $k_{s}$ in Fig. 3, it is observed that because in the $E_{0r} = E_{0s}$ case constructive interference occurs, a better result can be observed, but in other states, depending on which amplitude is more dominant over the other, the result is determined. Therefore, by selecting and filtering the appropriate amplitude, the resolution of holographic images can be increased or decreased. The vertical axis will represent the contrast or quality of the interference fringes, which can be described by Eq. 15, the interference intensity. The figure illustrates how the contrast and stability of the interference fringes depend on the ratio of the reference and sample beam amplitudes. When the amplitudes are comparable, constructive interference maximizes fringe visibility and holographic fidelity. In contrast, dominance of one beam suppresses interference contrast, reducing the effective phase sensitivity of the holographic reconstruction. This highlights the critical role of amplitude balancing in optimizing plasma-based holographic imaging.

**Fig. 4:**
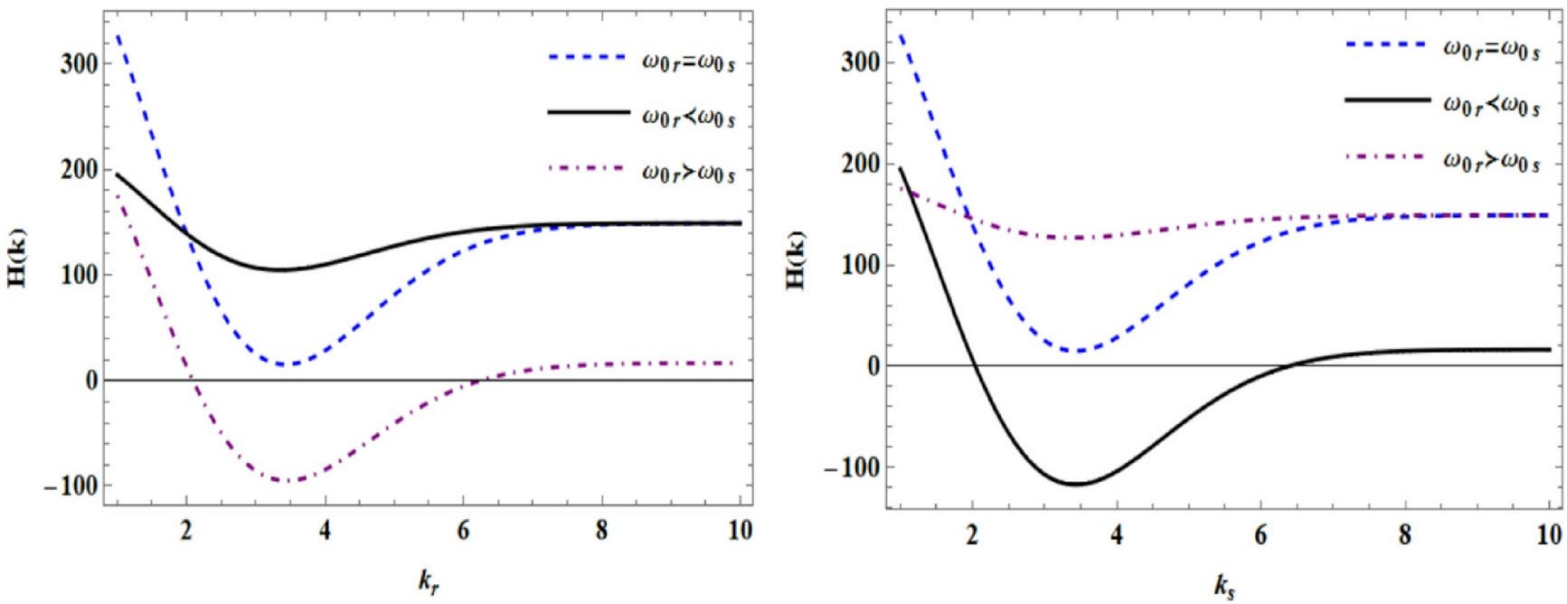
Holographic (quality of the interference fringes) diagram in terms of $k_{r}$ and $k_{s}$ with different Gaussian widths for both input waves.

In the Fig. 4, the holographic diagram is plotted in terms of $k_{r}$ and $k_{s}$ with different Gaussian widths for both input laser beams. The results show that beam waist parameters strongly influence the spatial-frequency filtering of the interference pattern and the resulting ponderomotive refractive-index modulation. Narrower beam waists enhance high-spatial-frequency components and improve holographic resolution, whereas broader beams lead to smoother interference patterns and reduced sensitivity to fine plasma structures. The transition from destructive to constructive interference with increasing wave number is clearly observed, demonstrating the interplay between beam geometry and holographic phase encoding in plasma.

In both diagrams, when $\omega_{0r} = \omega_{0s}$ is, it initially leads to a rapid decreasing slope due to destructive interference at lower frequencies, but then increases again at higher frequencies and reaches stability. In the case that $\omega_{0r} \prec \omega_{0s}$ at frequency $k_{r}$ the decreasing slope is very small and the holographic amplitude has an acceptable value, but in $k_{s}$ because the $\omega_{0s}$ component dominates, we observe the opposite result. With increasing frequency, the holographic amplitude tends to flatten and long-wave number features become less distinct. In the case of $\omega_{0r} \succ \omega_{0s}$, we also observe exactly all these changes. To exploit frequency details, the Gaussian width of both waves must be appropriately selected or a suitable filter must be applied so that important amplitudes are preserved.

Beyond its relevance as a theoretical framework, the present approach suggests potential applications in plasma diagnostics and applied optical systems. Because the holographic signal is directly linked to refractive-index modulations induced by ponderomotive effects, the technique may be used as a non-invasive diagnostic tool for probing density structures and phase variations in dynamic plasmas. Such capability is particularly attractive in high-intensity or short-duration plasma environments where conventional solid optical components are prone to damage or distortion. From an applied perspective, plasma-based holographic lenses may also find use in adaptive beam shaping or energy steering in high-power laser systems, where the absence of fixed material optics provides additional flexibility. While practical implementation would require careful control of plasma stability and phase coherence^30,31^.

## Conclusion

Ponderomotive plasma lenses provide a promising platform for plasma-based holography and dynamic wave-front control. In this work, we have presented a theoretical framework for holography based on the interference of two Gaussian laser beams, with explicit definition of the holographic geometry and the physical role of each beam. The analysis shows that plasma-induced refractive-index modulation can be effectively controlled through interference conditions, particularly via the transverse widths of the Gaussian inputs. We find that the holographic response depends on the wave number and relative beam amplitudes, with transitions between destructive and constructive interference influencing fringe stability and phase sensitivity. Appropriate adjustment of beam widths and operating parameters improves the coherence and spatial-frequency content of the interference pattern, leading to enhanced spatial resolution in the reconstructed refractive-index distribution. Although the present study is theoretical, it employs experimentally accessible laser parameters and provides a consistent basis for future numerical and experimental investigations of plasma-based holography.

## Data Availability

The data supporting the findings of this study are derived from analytical calculations and are fully presented within the manuscript. No additional raw datasets were generated.
